# Removal of Volatile Organic Compounds by Marine Sponges: Implications for Coastal Bioremediation

**DOI:** 10.1021/acs.est.5c05458

**Published:** 2025-12-16

**Authors:** Rafel Simó, Rafel Coma, Pau Cortés-Greus, Marta Masdeu-Navarro, Teresa Morganti, Marta Ribes

**Affiliations:** ‡ 58341 Institut de Ciències del Mar (ICM)−Consejo Superior de Investigaciones Científicas (CSIC). Passeig Marítim de la Barceloneta 37-49, 08003 Barcelona, Catalunya, Spain; § 54399 Centre d’Estudis Avançats de Blanes (CEAB)−Consejo Superior de Investigaciones Científicas (CSIC), Accés Cala Sant Francesc 14, 17300 Blanes, Girona, Catalunya, Spain

**Keywords:** volatile organic compounds, marine sponges, coastal areas, bioremediation, halomethanes, sulfur

## Abstract

Sponges (Porifera) are among the oldest known animal–microbe symbioses and are key players in marine biogeochemical cycles. Ubiquitous across benthic marine habitats, they process dissolved organic matter and participate in chemosynthetic pathways. We quantified, for the first time, volatile organic compounds (VOCs, namely, halomethanes, sulfur-containing compounds, and isoprene) in the inhaled and exhaled water of three Mediterranean sponge species: two high-microbial abundance (HMA), *Aplysina aerophoba* and *Agelas oroides*, and one low-microbial abundance (LMA), *Dysidea avara*. Using the Vacusip-INEX method in aquaria and *in situ* in a NW Mediterranean marine-protected area, we found that HMA sponges efficiently removed bromomethanes and dimethyl disulfide (DMS) and less clearly iodomethanes, carbon disulfide (CS_2_), and isoprene, while the LMA species removed methyl iodide (CH_3_I), DMS, and isoprene depending on the ambient seawater concentration. In preliminary experiments with *A. aerophoba* specimens, chemical inhibition of nitrification (with nitrapyrin) arrested bromomethane, DMS removal, and nitrate production, consistent with co-metabolic consumption by endosymbiotic nitrifying bacteria. Sponge VOC removal rates exceeded those of bacterioplankton by orders of magnitude. These findings underscore sponges as major sinks for VOCs in sponge-rich littoral ecosystems, with potential for bioremediation and mitigation of coastal VOC emissions, and call for further research into the ecological implications, impact on coastal air quality, and contributions to elemental cycling.

## Graphical abstract

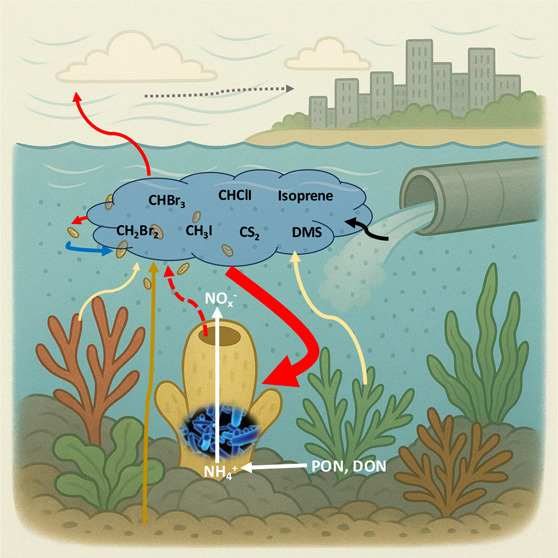

## 1 Introduction

Marine sponges (phylum Porifera) are among the oldest living multicellular animals, with fossils dating back 650 million years.^1^ Over 9000 known species inhabit diverse aquatic environments, from deep seas to tropical reefs, where they play critical ecological roles.^2^ Their efficient water-pumping, up to 28 000 L per liter of sponge per day,^3,4^ and their symbiotic relationships with diverse microbes^5^ underpin their role in biogeochemical cycling. Sponges use a varied array of nutritional strategies including photosynthesis, chemosynthesis and even carnivory.^6^ A key feature of their metabolism is their consumption of dissolved organic matter (DOM), which can comprise up to 97% of their diet,^7–14^ with removal rates proportional to the quality and concentration of DOM.^15^ However, the role of sponges in processing VOCs, short-lived reactive gases influential in air chemistry and climate, remains largely unknown.

Biogenic VOCs (BVOCs) are emitted by living organisms including humans.^16–18^ In aquatic systems, algal and coral BVOCs serve important eco-physiological functions, ranging from enhancing the emitters’ tolerance to abiotic and biotic stresses,^19^ to transferring important information between organisms. The latter includes the released of volatiles by algae as defenses against predation.^20^ Recently, the volatilome (the amount and speciation of VOCs) has been proposed as an indicator of coral reef health status.^17^

VOCs in aquatic systems also originate from anthropogenic sources. Some industrial activities and wastewater treatment plants dump volatile sulfur compounds into water bodies causing episodes of bad odors and potential impacts on human health and well-being.^21^ Chlorinated effluents from refrigeration, desalination, ballast water, and household wastewater are significant sources of volatile halocarbons in coastal waters,^22,23^ with largely unquantified effects on local ecosystems and air quality. This issue is especially relevant in coastal areas where terrestrial nutrient inputs may substantially enhance VOC emissions.^24^

The limited information we have so far indicates that shallow benthic marine communities, such as tropical coral reefs, are net producers of a number of VOCs^25^ even though some of these compounds are consumed by microorganisms at rates similar to or even greater than those of outgassing to the atmosphere.^26,27^

Given the role of demosponges as DOM consumers, and the diverse capabilities of their microbiomes,^28^ we hypothesized that sponges as holobionts may metabolize VOCs and influence their concentrations in coastal marine systems. To examine this, we analyzed three Mediterranean sponge species with contrasting habitat and harboring varying amounts of associated microbes: *Aplysina aerophoba*, a photophilic species that inhabits shallow, sunlit environments, and *Agelas oroides*, both classified as HMA species; the third was *Dysidea avara*, a LMA species that co-habits with *A. oroides* in the sciaphilic Mediterranean coralligenous community. We used the Vacusip-INEX method^29^ to compare VOC composition and concentration in the incoming (inhaled, IN) and outcoming (exhaled, EX) waters pumped by the sponges. This investigation expands our understanding of sponges’ functions in littoral ecosystems by adding roles in regulating the local info-chemical seascape and the emission of atmospheric reactive gases. It also points to their potential in the bioremediation of noxious compounds. Furthermore, it emphasizes the importance of protecting coastal areas where these communities reside, in order to preserve the ecosystem services they provide.

## 2 Materials and Methods

### 2.1 Organism Collection for Aquarium Experiments and *In Situ* Measurements

Specimens of *A. aerophoba* (*n* = 8), *A. oroides* (*n* = 8), and *D. avara* (*n* = 6) were collected in late August by SCUBA diving off the coast of Girona, Spain (42° 06′ 55″ N, 3° 10′ 8′′ E), at depths ranging from 3 to 15 m. The specimens were then transferred to the experimental aquaria zone (ZAE) at the Institute of Marine Sciences (ICM–CSIC) in Barcelona. They were housed in a 150 L water table supplied with a continuous flow of seawater at 50 L h^–1^. The seawater was pumped from an intake located 300 m offshore at a depth of 10 m and filtered through a sand filter. Sponge holobionts were acclimated for 5 days to allow the host and microbiota to adjust to the new environmental conditions.^30^ After acclimation, only actively pumping individuals, were used for experiments (*A. aerophoba* = 6, *A. oroides* = 7 and *D. avara* = 4). Each sponge was transferred to a 6 L aquarium supplied with fresh flowing seawater at a rate of ∼30 L h^–1^ for Vacusip-INEX measurements. All individual aquaria were kept together in a water bath at a constant temperature of 22 °C.

At the same site where the aquarium organisms have been previously collected, specimens of *A. aerophoba* (*n* = 5), *A. oroides* (*n* = 6), and *D. avara* (*n* = 6) were selected for *in situ* measurements in September. These specimens were chosen for their potential suitability for the Vacusip-INEX technique (see Morganti et al.^29^) for full methodological details]. Preference was given to specimens with large oscula and proper positioning on the substrate.

Ethical approval was not required for this study, as the research was conducted on marine sponges (phylum Porifera), which are invertebrate organisms not covered by Spanish Royal Decree 53/2013 or EU Directive 2010/63/EU on the protection of animals used for scientific purposes. All sampling was performed in a manner designed to minimize environmental impact. Fieldwork was conducted in the “Montgrí, Illes Medes i Baix Ter” Natural Park under permits 2020PNATMBTAUT010, 2021PNATMBTAUT0112, and 2022PNATMBTAUT0101. The facilities used for the aquarium experiments (Experimental Aquaria Zone-ZAE) hold the REGA (General Registry of Livestock Holdings) number ES080190036532, ensuring compliance with animal welfare regulations.

### 2.2 Vacusip-INEX Measurements

We followed the methodology described by Morganti et al.^29^ for the INEX VacuSIP method. Briefly, net fluxes of VOCs were determined by measuring concentration differences between the water inhaled and exhaled by the sponge. Exhaled water (EX) was directly sampled from the sponge osculum, while inhaled water (IN) was measured a few cm away, in both cases using a custom setup that employed vacuum pressure to draw in water. The sampling rate (<1 mL min^–1^) was kept sufficiently below the sponge pumping rate to prevent contamination of the exhaled sample with ambient water. Samples were collected in 40 mL certified cleaned-EPA vials. Samples were filtered using in-line stainless steel 13 mm Swinney filter holders installed with precombusted (400 °C for at least 2 h) binder-free glass fiber filters (GF/F 97% retention efficiency at 0.7 μm). Due to methodological constrains, it was not possible to completely fill the vials, leaving some headspace; however, previous trials confirmed the feasibility of measuring volatile compounds under these conditions. The vials were not further manipulated until their analysis (see below), which occurred within 4 h of collection.

### 2.3 Pumping Rate Measurements and VOC Fluxes

The sponge pumping rate (*P*_sponge_) was determined using a modification of the dye front speed method (DFS) introduced by Yahel et al.^31^ and detailed in Morganti et al.^32^ In short, a transparent tube was positioned as close as possible above the sponge osculum, and the movement of the dye inside the tube was recorded by a diver using a video camera. To minimize interference with the animals’ behavior and prevent deviations from ambient water density, sodium fluorescein powder was mixed with ambient water drawn into the syringe just before the recording. A disposable syringe filter (25 mm, 0.2 μm) was installed on the syringe to prevent the release of dye particles. The speed of the dye front inside the tube was measured through frame-by-frame analysis. The rate of water flow from the osculum was calculated following the method of Yahel et al.^31^ as the product of the tube’s cross-sectional area and the dye front speed. In the few cases where the tube was smaller than the osculum, the rate was calculated as the product of dye front speed and osculum’s cross-sectional area. Eight to ten replicates were conducted per specimen. The VOC mass flux (VOC_flux_, nmol min^–1^ cm^–2^ sponge) mediated by each sponge species was calculated by multiplying the mean ΔVOC_EX–IN_ (nmol L^–1^) by the mean sponge planar surface-specific pumping rate P_sponge_ (ml min^–1^ cm^–2^ sponge) of the examined specimens.

$${\mathrm{VOC}}_{\mathrm{flux}}=\Delta {\mathrm{VOC}}_{\mathrm{EX-IN}}\times P_{\mathrm{sponge}}$$

The water transit time through each sponge (s^–1^) was estimated by dividing the sponge’s volume (cm^3^) by its pumping rate (mL min^–1^). Each sponge specimen was photographed, and its height was measured both in the field and in the aquarium to estimate the sponge’s size and the dimensions of the osculum.

### 2.4 VOC Analysis

For VOC analyses, we employed an Agilent 5975T LTM gas chromatograph–mass spectrometer coupled with a Stratum (Teledyne Tekmar) purge and trap system.^25^ Seawater aliquots (15–25 mL) were withdrawn from the EPA vials with an Artiglass syringe equipped with a PTFE tube. After eliminating the air bubbles, the PTFE tube was removed, and the samples were injected into the purge vessel while filtered through a gas fiber filter (GF/F; 97% retention efficiency at 0.7 μm). VOCs were sparged at room temperature for 12 min with a flow rate of 40 mL/min of ultrapure helium, trapped on a VOCARB 3000 absorption column maintained at room temperature, and desorbed by heating to 250 °C. VOCs were separated using a capillary column LTM DB-VRX (Agilent; 20 m × 0.18 mm × 1 μm) held at 35 °C for 4 min, then ramped to 230 °C at 30 °C/min, and held at 230 °C for 4 min, resulting in a total analysis time of 14.5 min. The helium carrier gas flow rate was 0.8 mL min^–1^. Compounds were detected using an electron impact ionization mass spectrometer in selected ion monitoring mode. Target compounds included halocarbons [iodomethanes: methyl iodide (CH_3_I) and chloroiodomethane (CH_2_ClI); bromomethanes: dibromomethane (CH_2_Br_2_) and bromoform (CHBr_3_)]; sulfur compounds [dimethyl sulfide (DMS, CH_3_SCH_3_) and carbon disulfide (CS_2_)], and isoprene (C_5_H_8_). Compounds were identified by matching the retention times of their most characteristic (quantification) ions and their confirmation ions with those of pure standards.

### 2.5 Statistical Analyses

To test for significant differences in compound concentration between sites (*in situ* and aquaria facility), data were log-transformed to normalize the distribution. When normality (Shapiro test) and homogeneity of variances (Barlett test) were met, a two-sample *t* test was applied. When the assumption of equal variances was violated (heterogeneity of variances), a Welch two-sample *t* test was used.

Net removal or excretion of each compound was calculated by the difference in concentration between the exhaled and the inhaled water (ΔVOC_EX–IN_) for each sample pair. Positive values indicate excretion, while negative values indicate removal. To test for significant removal or excretion (ΔVOC_EX–IN_ ≠ 0), we used a paired *t* test unless the number of available pairs was small or data violated the normality assumption. In such cases, the non-parametric Mann–Whitney rank sum test was applied. Least-squares regression analysis was used to test the dependence of the ΔVOC_EX–IN_ on their ambient abundance. Data analysis was conducted using the statistical software R, version 4.4.2.

### 2.6 Nitrification Inhibition Experiments with Nitrapyrin

The oxidation of ammonium, the first of two steps of nitrification, was inhibited by the addition of nitrapyrin [2-chloro-6-(trichloromethyl)­pyridine]. A stock solution of 50 mg of nitrapyrin was prepared by mixing it with 1 L of 0.2 μm filtered seawater and allowing it to dissolve for 2 h. The resulting dilution was then added to the aquarium at a ratio of 1 part solution to 20 parts aquarium water, achieving a final concentration of approximately 1.5 mg/L (around 5 μmol/L) of nitrapyrin. A concentration of approximately 1 μM has been reported to inhibit ammonia oxidation in cultures of nitrifying bacteria.^33,34^ Three specimens of *A. aerophoba* were used for the nitrapyrin additions. Samples for inorganic nitrogen [nitrite plus nitrate (NO^–^_*x*_) and ammonia NH_4_^+^] were filtered using in-line stainless-steel 13 mm Swinney filter holders installed with precombusted (400 °C for at least 2 h) binder-free glass fiber filters (GF/F 97% retention efficiency at 0.7 um). Concentrations were measured with an Alliance autoanalyzer following the method of Grasshoff et al.^35^

### 2.7 Sponge Abundance Assessment

The assessment of the sponge abundance *in situ* was conducted using the point-intercept method and recent guidelines.^36^ Fifteen 10 m line transects widely distributed at random across the study area (∼12 000 m^2^) were laid down by divers at 20 m depth on the coralligenous community. Sampling intercepts was established as points along each transect at 0.2 m intervals (*N* = 50 intercepts/transect). Taxa were classified to the species level for sponges and to phyla for the other organisms of the benthic community at each intercept. The proportion of intercepts where a species was found was expressed as a percentage of the total intercepts sampled.

Then, the coverage of each sponge species (*L*) was estimated using the formula

$$L=\frac{L_{i}}{N}\times 100$$

where *L* is the percentage cover of the sponge species, *L*_i_ is the number of intercepts where a sponge species was encountered, and *N* is the total number of possible intercepts. As *N* increases, the ratio *L*_i_/*N* asymptotically approaches the actual cover value.

## 3 Results and Discussion

### 3.1 VOC Concentrations

VOC concentrations in both the *in situ* and aquaria settings were determined in the water inhaled by the sponges during the study period. At both locations, log-transformation normalized the distribution of most compounds, except for CH_2_Br_2_. The Bartlett test revealed heterogeneity of variance for CHBr_3_, CH_3_I, and CS_2_; therefore, a Welch two-sample test was performed for these compounds. Significant differences were observed in the concentration of CS_2_ and dimethyl sulfide DMS between the aquaria and *in situ*. CS_2_ levels were higher in the aquaria facility, while DMS had higher concentration *in situ*. Additionally, CH_2_ClI was exclusively detected in the aquaria (Table 1).

**Table 1:** Testing for Significant Differences in Ambient VOC Concentration between *In Situ* and the Aquarium Facility^a^

	*in situ*		aquaria facility		*t* test		
VOCs	*M* ± SD	*n*	*M* ± SD	*n*	*t*	df	*p*
CH_2_Br_2_ (pmol L^–1^)	5.7 ± 3.1	17	15.0 ± 20.7	19	–1.67	34	0.105
CHBr_3_ (pmol L^–1^)^b^	52.4 ± 21.7	17	54.1 ± 53.7	20	1.09	28	0.287
CH_2_CII (pmol L^–1^)	nd		2.1 ± 2.1	20	nd		
CH_3_I (pmol L^–1^)^b^	4.0 ± 1.1	17	7.2 ± 9.3	20	0.50	21	0.624
CS_2_ (pmol L^–1^)^b^*	28.6 ± 12.5	17	53.8 ± 34.1	20	–3.12	31	**<0.01**
DMS (nmol L^–1^)*	1.4 ± 0.5	17	0.7 ± 0.5	20	5.00	35	**<0.0001**
isoprene (pmol L^–1^)	24.5 ± 7.8	17	26.6 ± 12.2	20	–0.23	35	0.820

a *M*, mean; SD, standard deviation; *n*, sampling size; *t*, *t* statistic; df, degrees of freedom; *p*, probability value; and nd, not detected. Significant differences are shown in bold.

b The Welch test is reported when Bartlett′s test indicated that the homogeneity of variance assumptions was not met for this variable.

While the study was not originally designed to compare VOC concentrations across sites, the distinct environmental conditions of the two locations led to notable differences. The ZAE facility at the ICM–CSIC, Barcelona, is supplied with seawater through a pipeline whose inlet is located near the coastline, at a depth of 10 m. In contrast, the field site is located in a protected marine area 140 km north of Barcelona and 1 nautic miles offshore. The elevated CS_2_ levels and exclusive detection of CH_2_ClI in the aquaria point toward anthropogenically enhanced sources, aligning with previous findings.^37^ These results suggest a combination of natural and human-derived inputs in the aquarium water. In contrast, higher concentrations of DMS *in situ*, particularly at the photophilic site where *A. aerophoba* was examined, suggest a primarily biogenic origin, likely from phytoplankton and benthic photoautotrophs.

VOCs in aquatic environments originate from two principal sources: biogenic emissions and anthropogenic activities. BVOCs are emitted by living organisms as metabolic byproducts or intermediates, whereas anthropogenic sources include industrial discharge and chlorination of domestic and industrial wastewater.^16–18^ Some VOCs produced anthropogenically coincide with BVOCs, potentially disrupting their ecological roles as infochemicals, antimicrobial agents, antioxidants, or allelochemicals^17,18^ and references therein.

### 3.2 Assessing VOC Removal by Marine Sponges

To assess the removal or excretion of VOCs by marine sponges, we applied two complementary approaches: testing whether ΔVOC_EX–IN_ ≠ 0, and conducting regression analysis between ΔVOC_EX–IN_ and ambient concentration (see the Materials and Methods for details). Although both methods yielded consistent results in most cases, discrepancies occasionally occurred. In some cases, ΔVOC_EX–IN_ was different from zero while the regression was not significant (e.g., CHBr_3_, CH_2_ClI, and CH_3_I in *A. oroides*; isoprene in *A. aerophoba*; Figures 1 and 2 and Table 2), or the opposite, ΔVOC_EX–IN_ was not significantly different from zero while the regression was significant (e.g., CH_3_I, DMS, and isoprene in *D. avara*; and isoprene in *A. oroides*; Figures 1 and 2 and Table 2). This mismatch is attributed to the concentration-dependent nature of removal. Sponges may require a threshold ambient concentration above which they efficiently remove a compound (see the results below). Therefore, the number of data pairs falling below or above this concentration threshold can influence whether or not ΔVOC_EX–IN_ is significantly different from zero. The present study examined the effect of sponges on VOCs under natural concentrations. However, for some compounds, this was limited by a rather narrow range of concentrations. This is because the significance of the regression depends on the range of natural concentrations encountered during the experiments; broader concentration ranges, such as those observed across different seasons, typically enhance regression significance.^15^ Due to this limitation, in our experiments we considered the removal of a compound to be significant if either the regression was statistically significant or the concentration difference ΔVOC_EX–IN_ was significantly different from zero. Based on these criteria, all the analyzed compounds were found to be significantly removed by at least one sponge species (see below).

**Figure 1:**
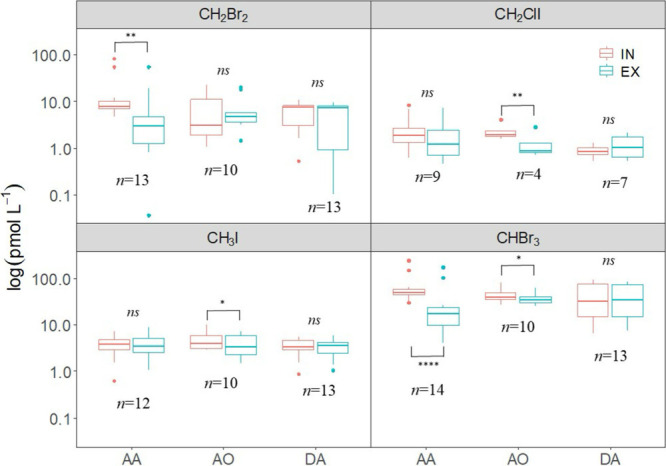
Inhaled (IN, red) and exhaled (EX, blue) concentrations of CH_2_Br_2_, CHBr_3_, CH_2_CII, and CH_3_I in the three assayed sponge species: (AA, *A. aerophoba*; AO, *A. oroides*; and DA, *D. avara*) combining data from *in situ* and aquaria samples. The sample size (*n*) is indicated for each IN-EX pair. ns, *p* > 0.05; ∗, *p* ≤ 0.05; ∗∗, *p* ≤ 0.01; ∗∗∗, *p* ≤ 0.001; and ∗∗∗∗, *p* ≤ 0.0001.

**Figure 2:**
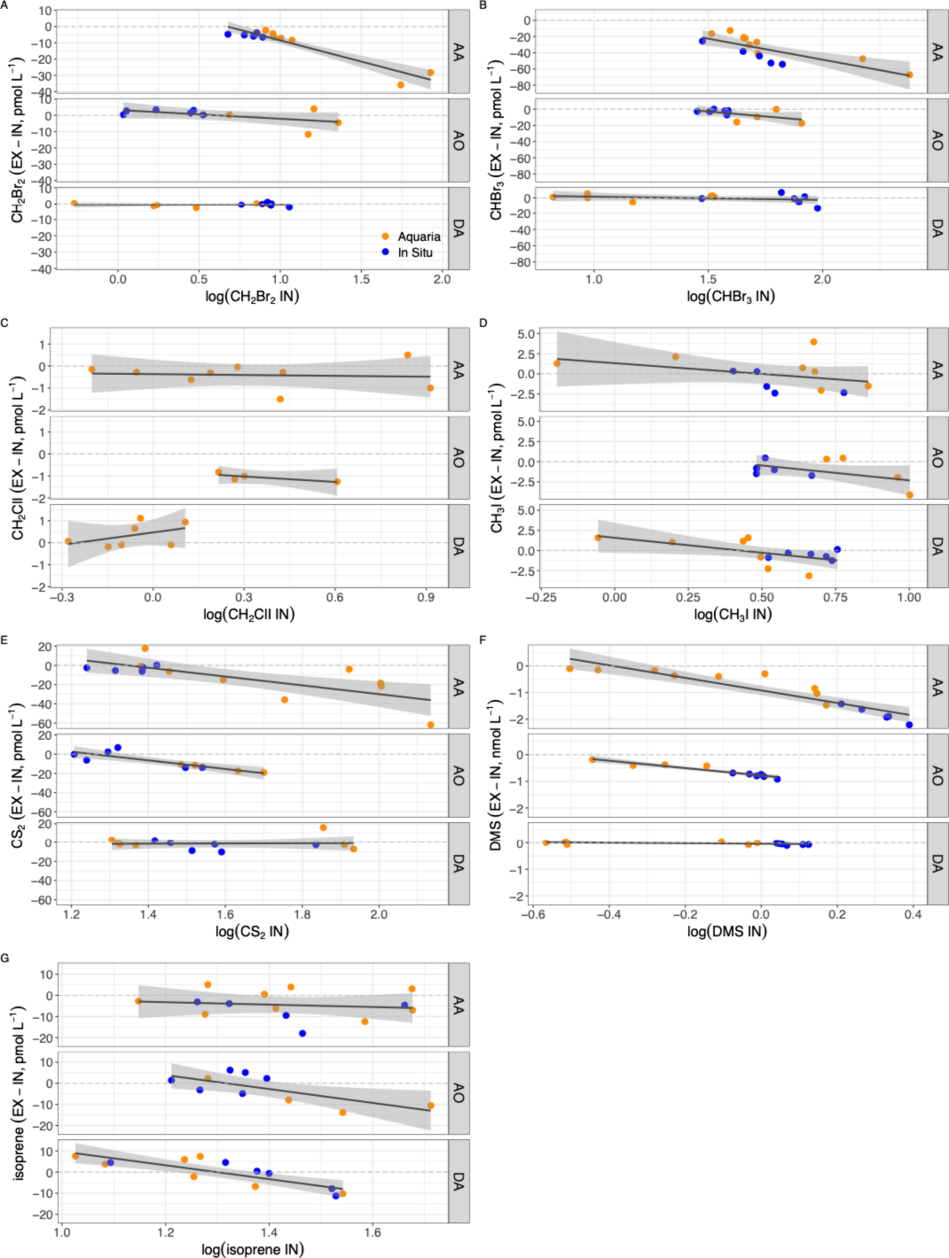
Relationship between the net removal (or excretion) of volatiles by three Mediterranean sponge species and the ambient concentration from *in situ* (blue) and aquaria (orange) samples. Each panel shows one compound: (A) CH_2_Br_2_, (B) CHBr_3_, (C) CH_2_CII, (D) CH_3_I, (E) CS_2_, (F) DMS, and (G) isoprene. The *y* axis represents the difference between inhaled and exhaled VOC concentrations, where negative values indicate removal and positive values indicate excretion by the sponges. Note that ambient concentrations on the *x* axis are log-transformed (AA, *A. aerophoba*; AO, *A. oroides*; and DA, *D. avara*). The gray shading indicates 95% CI.

**Table 2:** Parameters of the Regression Lines between the Net Removal (or Excretion) and Ambient Concentrations for All Analyzed Compounds and Sponge Species^a^

	*A. aerophoba*			*A. oroides*			*D. avara*		
VOCs	*a*	*b*	*R* ^2^	*a*	*b*	*R* ^2^	*a*	*b*	*R* ^2^
CH_2_Br_2_ (pmol L^–1^)	–2.62 ± 1.54	–0.39 ± 0.05	0.83***	2.37 ± 1.86	–0.34 ± 0.18	0.30	–0.83 ± 0.67	0.03 ± 0.10	0.01
CHBr_3_ (pmol L^–1^)	–21.97 ± 4.99	–0.20 ± 0.06	0.51**	3.50 ± 5.42	–0.21 ± 0.11	0.30	2.57 ± 2.42	–0.07 ± 0.05	0.17
CH_2_CII (pmol L^–1^)	–0.40 ± 0.32	–0.00 ± 0.08	0.00	–0.77 ± 0.20	–0.13 ± 0.08	0.56	–0.49 ± 0.79	0.93 ± 0.85	0.19
CH_3_I (pmol L^–1^)	1.59 ± 1.32	–0.43 ± 0.31	0.16	0.61 ± 0.84	–0.33 ± 0.15	0.38	1.64 ± 0.96	–0.54 ± 0.23	0.31*
CS_2_ (pmol L^–1^)	7.44 ± 5.71	–0.38 ± 0.09	0.59**	11.47 ± 4.42	–0.67 ± 0.14	0.74**	–2.27 ± 3.90	0.02 ± 0.08	0.005
DMS (nmol L^–1^)	0.35 ± 0.1	–1.04 ± 0.07	0.95***	0.11 ± 0.07	–0.91 ± 0.08	0.94***	0.03 ± 0.03	–0.07 ± 0.03	0.32*
isoprene (pmol L^–1^)	–2.84 ± 5.07	–0.06 ± 0.16	0.01	0.11 ± 0.07	–0.91 ± 0.08	0.94***	15.24 ± 2.79	–0.71 ± 0.12	0.76***

a The regression lines are represented in Figure 2. *a*, intercept ± standard error; *b*, regression line coefficient ± standard error; and *R* ^2^, coefficient of determination. ∗, *p* ≤ 0.05; ∗∗, *p* ≤ 0.01; and ∗∗∗, *p* ≤ 0.001.

### 3.3 Halomethanes

Among halomethanes, CH_2_Br_2_ showed a significant decrease in mean exhaled concentration compared to inhaled water, indicating removal by *A. aerophoba*, (Figure 1A and Table S1). These removal of CH_2_Br_2_ is shown by most of the pairs (12 out of 13) in *A. aerophoba* whereas the other two sponge species, *A. oroides* and *D. avara*, showed pairs with either removal or production (Figure S1) that resulted in mean ΔVOC_EX–IN_ not different from zero (Figure 1A, Table S1). CH_2_Br_2_ removal by *A. aerophoba* was significantly related to its concentration in the water column (Figure 2A and Table 2). CHBr_3_ showed significant mean removal by both HMA species, *A. aerophoba* and *A. oroides* (Figure 1B and Table S1), with all pairs showing a clear removal in *A. aerophoba* but few pairs showing removal in *A. oroides*. A mixture of removal and production pairs were found in *D. avara* (Figure S1). CHBr_3_ removal by *A. aerophoba* exhibited a linear relationship with water concentration (Figure 2B and Table 2). The CH_2_ClI and CH_3_I showed significantly non-zero difference between exhaled and inhaled concentrations only in *A. oroides* (Figure 1C and D and Table S1), with most replicate pairs exhibiting a consistent pattern (Figure S2). In contrast, the other two species displayed a mix of outcomes, including removal, excretion or no significant change (Figure S2). No significant correlation was found between CH_2_ClI removal and its concentration in the water (Figure 2C and Table 2). Conversely, for CH_3_I, despite the lack of a significant ΔVOC_EX–IN_ from zero, all sponge species showed a trend toward increased removal with rising water concentration. However, this trend reached statistical significance only in *D. avara* (Figure 2D and Table 2).

The main natural processes governing halomethane production in marine environments are the microbial methylation of a halogen atom, which leads to, e.g., CH_3_I,^38^ and the reaction of the enzymes haloperoxidases with hydrogen peroxide and dissolved organic matter, which leads to, e.g., the generation of bromomethanes by seaweeds and phytoplankton.^39,40^ Halocarbon production has been documented to serve ecological roles such as chemical defense against predators or competition with other organisms. In addition to the natural processes, anthropogenic activities, including halogenation of waters in water treatment plants and industrial cooling systems as well as desalination plant effluents^41^ and references therein, are also a source of halomethanes to coastal waters. Identifying how volatile halocarbons are removed is essential to improve our understanding of the marine carbon and halogen cycles as well as to design bioremediation solutions. To our knowledge, most of the research on the role of benthic organisms in halocarbon cycling has focused on the production of these compounds by primary producers,^42^ and references therein, while the potential of benthic holobionts to remove and metabolize halocarbons has been largely overlooked. Our study demonstrates that sponges remove bromo- and iodomethanes, with removal rates positively correlated with halomethane concentration. This indicates that sponge holobionts act as effective bioremediators of halomethanes, particularly bromomethanes, likely shaped by coevolution between microbial symbionts and their host in response to naturally occurring halocarbons.

### 3.4 Sulfur Volatiles

Among sulfur compounds, CS_2_ underwent removal by most of the sample pairs of *A. aerophoba* and *A. oroides* (Figure S3), with ΔVOC_EX–IN_ values significantly different from zero (Figure 3A and Table S1). Additionally, a positive relationship was found between CS_2_ removal and ambient concentration (Figure 2E and Table 2). DMS was consistently removed by both HMA species, *A. oroides* and *A. aerophoba*, with all pairs showing the same pattern (Figure S3). ΔVOC_EX–IN_ was significantly different from zero in *A. oroides* and *A. aerophoba* (Figure 3B). In all sponge species, DMS removal increased significantly with ambient concentration (Figure 2F and Table 2).

**Figure 3:**
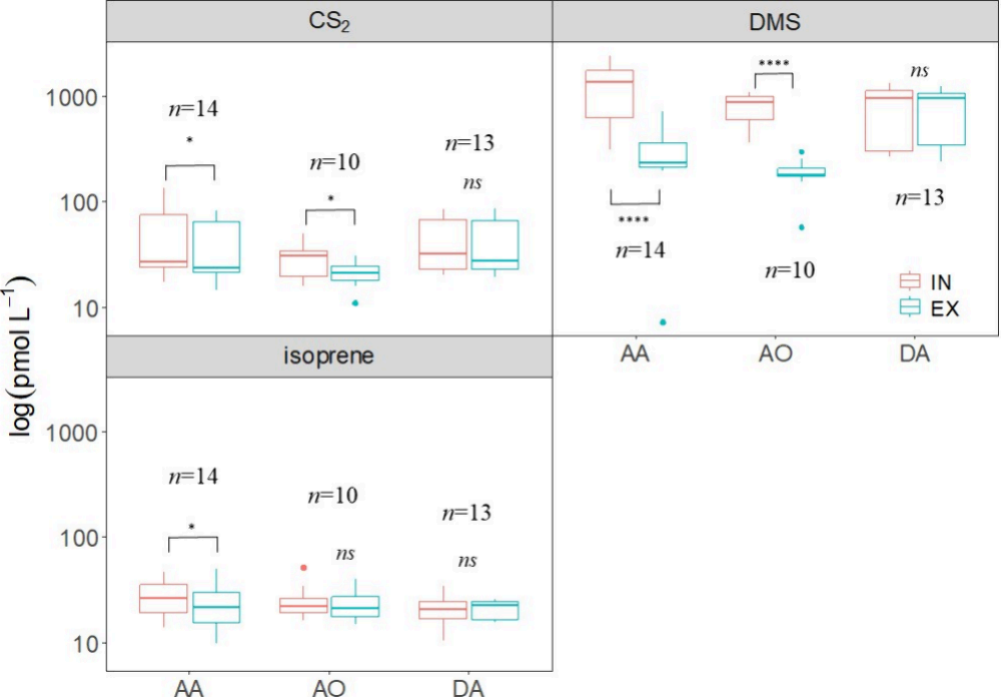
Inhaled (IN) and exhaled (EX) concentrations of (A) CS_2_, (B) DMS, and (C) isoprene in the three assayed sponge species (AA, *A. aerophoba*; AO, *A. oroides*; and DA, *D. avara*) combining data from *in situ* and aquaria samples. ∗, *p* ≤ 0.05; ∗∗, *p* ≤ 0.01; ∗∗∗, *p* ≤ 0.001; and ∗∗∗∗, *p* ≤ 0.0001.

Sulfur is an essential biological element that plays a crucial role in biogeochemical cycles, closely interconnected with the cycling of carbon, nitrogen, and phosphorus. The S cycle encompasses a wide range of chemical forms and redox states, driven by taxonomically diverse microbial assemblages. Understanding the functional processes and fluxes of various sulfur compounds offers valuable insights into broader biogeochemical interactions.^43^ Moreover, volatile sulfur compounds (VSCs) can be harmful to human health, affecting the respiratory system, eyes, and skin, and are often associated with unpleasant odors, contributing to their discomforting nature.^44^ In the marine realm, process studies have mainly focused on DMS, while the main sources and sinks of other VSC like CS_2_ remain poorly investigated. Besides, most of the published research deals with planktonic sources, e.g.,^18^ often overlooking the potential role of benthic filter feeders. Recent studies, however, suggest that benthic reef communities may act as hotspots for the cycling of a variety of VSCs.^42^ Our measurements reveal significant removal of DMS, and CS_2_, with HMA sponges acting as previously unrecognized sinks for CS_2_. This is a novel finding, as prior knowledge was largely limited to physicochemical and degassing processes as the main mechanisms for CS_2_ removal.^45^

### 3.5 Involvement of Sponge-Associated Nitrifiers in the Removal of Halomethanes and Sulfur Volatiles

Based on the observation that halomethanes and sulfur volatiles removal predominantly occurred in the two HMA sponge species, we suspected that sponge-mediated removal may arise from co-metabolism by nitrifying members of the sponge microbiota. Co-metabolism refers to the transformation of a non-growth substrate in the presence of a growth substrate, thereby broadening the range of organic compounds that can be degraded. This process occurs when enzymes, induced by a primary substrate, catalyze reactions involving other compounds that do not serve as energy or carbon sources for the microbe. This concept is particularly relevant in the context of VOCs and nitrification, where certain volatiles can be co-metabolically degraded alongside the nitrification process. Nitrification, a key microbial process in the nitrogen cycle, involves the stepwise oxidation of ammonia to nitrate and is widely recognized as a natural bioremediation mechanism.^46,47^ In the initial step, the enzyme ammonia monooxygenase (AMO) catalyzes the oxidation of ammonia to hydroxylamine, which is subsequently converted to nitrate by hydroxylamine oxidoreductase (HAO). Monooxygenases are known for their broad substrate specificity, including ammonia, methane and DMS, and can co-metabolically oxidize a range of secondary substrates, such as polyhalomethanes and sulfides.^48–51^ Marine sponges are well-established as important nitrifiers and play a critical role in nitrogen cycling in ecosystems where they are abundant.^52,53^ In sponges, nitrification is conducted by dense populations of endosymbiotic nitrifying bacteria.^53^ To investigate the link between nitrification and halomethane and organo-sulfide removal, we used nitrapyrin, a known inhibitor of nitrification,^33^ in experiments with *A. aerophoba*.

At 9.30 am, before the addition of nitrapyrin, an INEX measurement was performed to analyze ammonium, NO_*x*_^–^ (nitrite + nitrate), bromomethanes, and DMS (Figure 4). Specimens 1 and 2 removed ammonium and excreted NO_*x*_^–^, while individual 3 excreted both (Figure 4A and B). Additionally, prior to the nitrapyrin addition, all three individuals removed similar amounts of dibromomethane, variable amounts of bromoform, and DMS (Figure 4C–E). Following the nitrapyrin addition at 10:30 a.m., individuals 1 and 2 progressively decreased both ammonium removal and NO_*x*_^–^ production (Figure 4A and B), indicating inhibition of nitrification. Concurrently, their removal of bromomethanes and DMS declined to zero and even shifted to net production after 8 h (Figure 4C–E). In contrast, individual 3 continued excreting NO_*x*_^–^ at consistent rate for the first 6 h and at a significantly increased rate during the final 2 h (Figure 4B), while its ammonium excretion shifted to removal (Figure 4A). Thus, for reasons yet unknown, nitrapyrin did not inhibit nitrification in individual 3. Interestingly, this specimen continued to remove bromomethanes and DMS throughout the 8 h of incubation, regardless of the nitrapyrin presence (Figure 4C–E).

**Figure 4:**
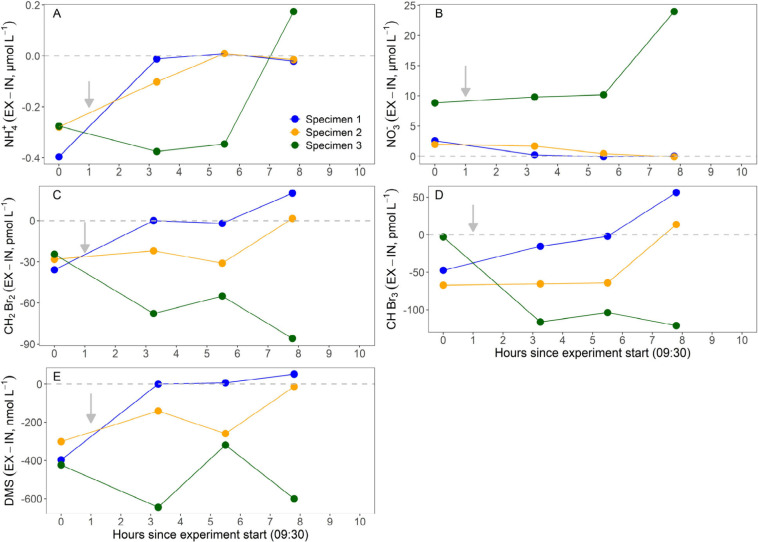
Nitrification inhibition experiments with nitrapyrin on three specimens of *A. aerophoba*. The *y* axis shows the difference between inhalant and exhalant concentrations of (A) NH_4_^+^, (B) NO_3_^–^, (C) CH_2_Br_2_, (D) CHBr_3_, and (E) DMS; the *x* axis shows time since nitrapyrin addition. The grey arrow indicates when nitrapyrin was added.

Although the experiment involved a small sample size (*n* = 3), successful nitrification inhibition was achieved in two of the specimens in which polyhalomethane and DMS consumption was arrested. Hence, the data are consistent with co-metabolic coupling, within the sponge holobiont, between nitrification and the degradation of CHBr_3_ ^51^ CH_2_Br_2_ ^54^ Results also suggest the involvement of nitrifying bacteria in DMS removal. Among the microbial processes that consume DMS in marine aerobic environments there is the oxidation to DMSO by monooxygenases like the ammonia monooxygenase (AMO) present in cultured marine strains of nitrifying bacteria.^55^ Therefore, the fact that nitrapyrin inhibited DMS loss indicates that most, if not all, DMS removal by sponges was due to oxidation by nitrifier-associated AMO. Note that the nitrapyrin addition assays indicate active microbial removal and argue against simple abiotic sorption to sponge matrices. Nevertheless, we use the conservative term “removal” rather than “consumption” until further mechanism-specific experiments provide definitive proof. All in all, our preliminary results support investigating the VOC bioremediation capabilities of marine sponges through co-metabolic processes of the prokaryotic endosymbionts. Here we show indication of the involvement of nitrifiers but also methanotrophs (and their methane monooxygenases) deserve attention.^48^

Our results also add to the notion of sponges as powerful models to dissect reductive dehalogenation. Large and aromatic organohalides are mostly respired by organohalide-respiring bacteria (OHRB) in anaerobic niches in sponges and sediments,^56–58^ with isotopic evidence for enzymatic debromination in sponge-derived bacterial cultures.^59^ In contrast, halomethanes, as the ones studied in this work, are suggested to be removed co-metabolically via monooxygenases in sponge holobionts.^28,60,61^ In either case, this is the first report for realistic removal rates under different conditions and species. Sponge microgradients may also foster abiotic reductions mediated by reduced sulfur/iron phases and organic redox mediators, complementing biological sinks.^57,62^ Together, sponges enable partitioning, and quantification, of respiratory, co-metabolic, and abiotic contributions to organo-halide loss.

### 3.6 Isoprene

Paired measurements of isoprene showed both removal and production events (Figure S4), resulting in no statistically significant deviation from zero in mean ΔVOC_EX–IN_ for *A. oroides* and *D. avara* (Figure 3C). However, regression analyses showed increased removal with increasing ambient isoprene concentration (Figure 2G and Table 2). Conversely, *A. aerophoba* exhibited significant isoprene removal (Figure 3C and Table S1) but unsignificant correlation between removal and ambient concentration (Figure 2G). Isoprene is a known non-methane biogenic volatile in marine environments. In surface waters, it is mainly produced by phytoplankton and seaweeds, with additional contributions from the photochemical degradation of dissolved organic matter under ultraviolet radiation and ozone deposition.^63^ Isoprene poses risks to human health primarily through its role in the formation of ozone and the generation of secondary organic aerosols, which can be of respiratory and cardiovascular concern.^24^ Since vascular plants and trees on land are major sources of isoprene to the atmosphere, the potential adverse effects of marine isoprene on air quality^24^ are more pronounced in coastal regions with high marine primary productivity and low terrestrial vegetation. Similar to other volatile compounds examined, our findings underscore a potentially role of benthic holobionts in regulating coastal marine isoprene emissions.

### 3.7 VOC Removal by Sponges vs Other Losses in Seawater

The removal rate constants of volatile compounds by the three sponge species were estimated by dividing the delta (ΔVOC_EX–IN_) of each compound by the transit time of inhaled water through the sponge, then dividing by the inhaled compound concentration. The obtained values were compared with existing data on bacterioplankton-mediated or chemical removal rate constants for each compound. In *A. aerophoba*, removal rates of CH_2_Br_2_ and CHBr_3_ reached ca. 140 h^–1^; these values are 3–6 orders of magnitude higher than the chemical and microbial loss rate constants reported for bromomethanes in the surface ocean 0.00001–0.02 h^–1^.^64,65^ For DMS, rate constants (164, 172, and 32 h^–1^ in *A. aerophoba*, *A. oroides*, and *D. avara*, respectively) were 2–3 orders of magnitude higher than the maximum bacterioplankton consumption rates previously reported for this compound in the surface ocean, 0.2 h^–1^.^66^ Similarly, sponge removal of CS_2_ (48 and 63 h^–1^ in *A. aerophoba* and *A. oroides*, respectively), isoprene (20, 15, and 33 h^–1^ in *A. oroides*, *D. avara*, and *A. aerophoba*, respectively), and CH_3_I (49 and 75 h^–1^ in *A. oroides* and *D. avara*, respectively) were at least 3 to 4 orders of magnitude times faster than the reported fastest loss rates in the surface ocean, 0.003, 0.001–0.03, and 0.00004–0.006 h^–1^ for CS_2_, isoprene, and CH_3_I, respectively.^26,45,67^ These results are consistent with the fact that prokaryotic abundances in HMA sponge tissues are orders of magnitude higher that abundances in seawater, and underscore the efficiency of sponge-mediated VOC removal.

### 3.8 Assessment of Sponge Holobionts as Benthic VOC Sinks

As an additional approach to evaluating the role of marine sponges as *in situ* bioremediators that regulate VOC concentrations and emissions in littoral ecosystems, we combined delta (ΔVOC_EX–IN_) values for various compounds with species-specific pumping rates per surface area (mL min^–1^ cm^–2^ sponge) and species cover in the sciaphilic coralligenous community (*D. avara* and *A. oroides*) and shallow photophilic zones [*A. aerophoba*,^68^]. Among halomethanes, iodomethanes and bromomethanes were removed by at least one sponge species, with the highest removal rate for CHBr_3_ by *A. aerophoba* at 0.1 μmol day^–1^ m^–2^ (Table 3). DMS was the sulfur compound most efficiently removed, particularly by the two HMA species, at rates of 1.3–3.2 μmol day^–1^ m^–2^, followed by the LMA species at 0.2 μmol day^–1^ m^–2^ (Table 3). Other VOCs, such as isoprene and CS_2_, were also removed at rates ranging from 2 to 15 nmol day^–1^ m^–2^ for isoprene and 19–38 nmol day^–1^ m^–2^ for CS_2_ (Table 3). While further data on community fluxes under varying conditions are needed, these results represent an initial estimate of coastal benthic sponges as sinks for volatiles.

**Table 3:** Assessment of VOC Consumption Fluxes by Mediterranean Sponges, Accounting for Their Benthic Cover^a^

	ΔVOC_EX–IN_ (×10^–6^ nmol mL^–1^)	*P*_sponge_ (mL min^–1^ cm^–2^ sponge)	sponge/benthic cover (cm^2^ m^–2^)	VOC flux (nmol day^–1^ m^–2^)
CH_2_Br_2_				
*A. aerophoba*	5.3 ± 1.8	15.2 ± 2.4	149	17 ± 6
CHBr_3_				
*A. oroides*	6.0 ± 6.3	14.2 ± 4.9	110	13 ± 14
*A. aerophoba*	32 ± 14	15.2 ± 2.4	149	104 ± 45
CH_2_ClI				
*A. oroides*	1.1 ± 0.2	14.2 ± 4.9	110	2 ± 0.5
CH_3_I				
*A. oroides*	1.1 ± 1.4	14.2 ± 4.9	110	2 ± 3
*D. avara*	0.3 ± 1.4	18 ± 3	230	2 ± 8
CS_2_				
*A. oroides*	8.4 ± 8.8	14.2 ± 4.9	110	19 ± 20
*A. aerophoba*	12 ± 19	15.2 ± 2.4	149	38 ± 62
DMS				
*A. oroides*	610 ± 200	14.2 ± 4.9	110	1372 ± 450
*D. avara*	30 ± 50	18 ± 3	230	179 ± 298
*A. aerophoba*	1000 ± 800	15.2 ± 2.4	149	3261 ± 2609
isoprene				
*A. oroides*	2.3 ± 6.7	14.2 ± 4.9	110	5 ± 15
*D. avara*	0.4 ± 6.8	18 ± 3	230	2 ± 41
*A. aerophoba*	4.6 ± 6.5	15.2 ± 2.4	149	15 ± 21

a *P*_sponge_, pumping rate; sponge/benthic cover, ratio of the surface area of individual sponge species to the total benthic surface area. Only compounds with ΔVOC_EX–IN_ ≠ 0 or a significant regression with ambient concentration were considered for each species.

### 3.9 Environmental Implications and Research Perspectives

Understanding the production, transport, and loss of VOCs in the marine environment is critical for elucidating ecosystem functioning and the biogeochemical cycling of elements like carbon, nitrogen, and sulfur, all of which include volatile forms. It is also critical for assessing oceanic impacts on human health. VOCs in coastal waters are receiving increasing attention due to concerns about how they alter atmospheric chemistry and pose risks to human health.^24^ Although VOCs are integral to ecosystem dynamics, elevated concentrations and emissions may cause odors, alter the oxidative capacity of the coastal atmosphere, and increase the concentration of hazardous air components like ozone. Human density-dependent eutrophication of littoral waters, together with the discharge of industrial and water treatment plant effluents, increase VOC sources in the coastal marine environment. Understanding the role of benthic filter feeding communities as sinks of VOCs is essential if we are to get any near to closing the budget of these compounds and developing mitigation strategies to their emissions.

Our findings reveal that high microbial abundance sponges efficiently remove a suite of VOCs of natural and anthropogenic origin, including sulfur-containing volatiles and polyhalomethanes. For these compound classes in particular, our results suggest the involvement of endosymbiotic nitrifying bacteria, which are common in sponges worldwide. Given the abundance of sponges in many littoral settings, their high pumping capacities, and the fact that removal rates by sponge holobionts are several orders of magnitude faster than those of bacterioplankton and chemical degradation, we conclude that sponges act as bioremediators for reactive volatiles that affect air quality. Further research is warranted to expand the suite of compounds screened for removal, elucidate the underlaying mechanisms, and incorporate hydrodynamics into community-scale flux measurements.

## Supplementary Material



## Notes

Testing for significant removal or excretion by assessing whether the difference between exhaled and inhaled concentrations is significantly different from zero (ΔVOC_EX–IN_ ≠ 0) (Table S1), detailed information about bromomethane concentrations in exhaled (EX) and inhaled (IN) waters in measurements conducted with the three sponges in aquaria and *in situ* (Figure S1), detailed information about iodomethane concentrations in exhaled (EX) and inhaled (IN) waters in measurements conducted with the three sponges in aquaria and *in situ* (Figure S2), detailed information about sulfur compound concentrations in exhaled (EX) and inhaled (IN) waters in measurements conducted with the three sponges in aquaria and *in situ* (Figure S3), and detailed information about isoprene concentrations in exhaled (EX) and inhaled (IN) waters in measurements conducted with the three sponges in aquaria and *in situ* (Figure S4) (PDF)
