# Supplementary material for: Removal of Volatile Organic Compounds by Marine Sponges: Implications for Coastal Bioremediation
Source: Environ Sci Technol. 2025 Dec 16;59(51):27869–80. doi: 10.1021/acs.est.5c05458 (PMC12756903; doi:10.1021/acs.est.5c05458)
Supplement: Supplementary file 1 [file es5c05458_si_001.pdf]

Removal of volatile organic compounds by marine sponges: implications for coastal bioremediation

Rafel Simó[1]‡, Rafel Coma[2], Pau Cortés-Greus[1], Marta Masdeu-Navarro[1], Teresa Morganti[1], Marta Ribes[1]‡**

[1] Institut de Ciències del Mar (ICM-CSIC). Passeig Marítim de la Barceloneta 37-49, 08003 Barcelona, Catalunya, ES

[2] Centre d'Estudis Avançats de Blanes (CEAB-CSIC), Accés Cala Sant Francesc 14, 17300 Blanes, Girona, Catalunya, ES

Corresponding Authors: *rsimo@icm.csic.es; *mribes@icm.csic.es

Supporting Information

Table S1: Testing for significant removal or excretion by assessing whether the difference between Exhaled and Inhaled concentrations is significantly different from zero ($\Delta VOC_{Ex\text{-}In} \neq 0$).

**VOCs**	*A. aerophoba*	*A. oroides*	*D. avara*
CH_2Br_2 (pmol L^{-1})	<0.01	ns	ns
$CHBr_3$ (pmol L^{-1})	<0.0001	0.015	ns
CH_2ClI (pmol L^{-1})[a]	ns	0.002	ns
CH_3I (pmol L^{-1})	ns	0.04	ns
CS_2 (pmol L^{-1})	0.04	0.014	ns
DMS (nmol L^{-1})	<0.0001	<0.0001	ns
Isoprene (pmol L^{-1})	0.02	ns	ns

[a]Only aquaria facility data

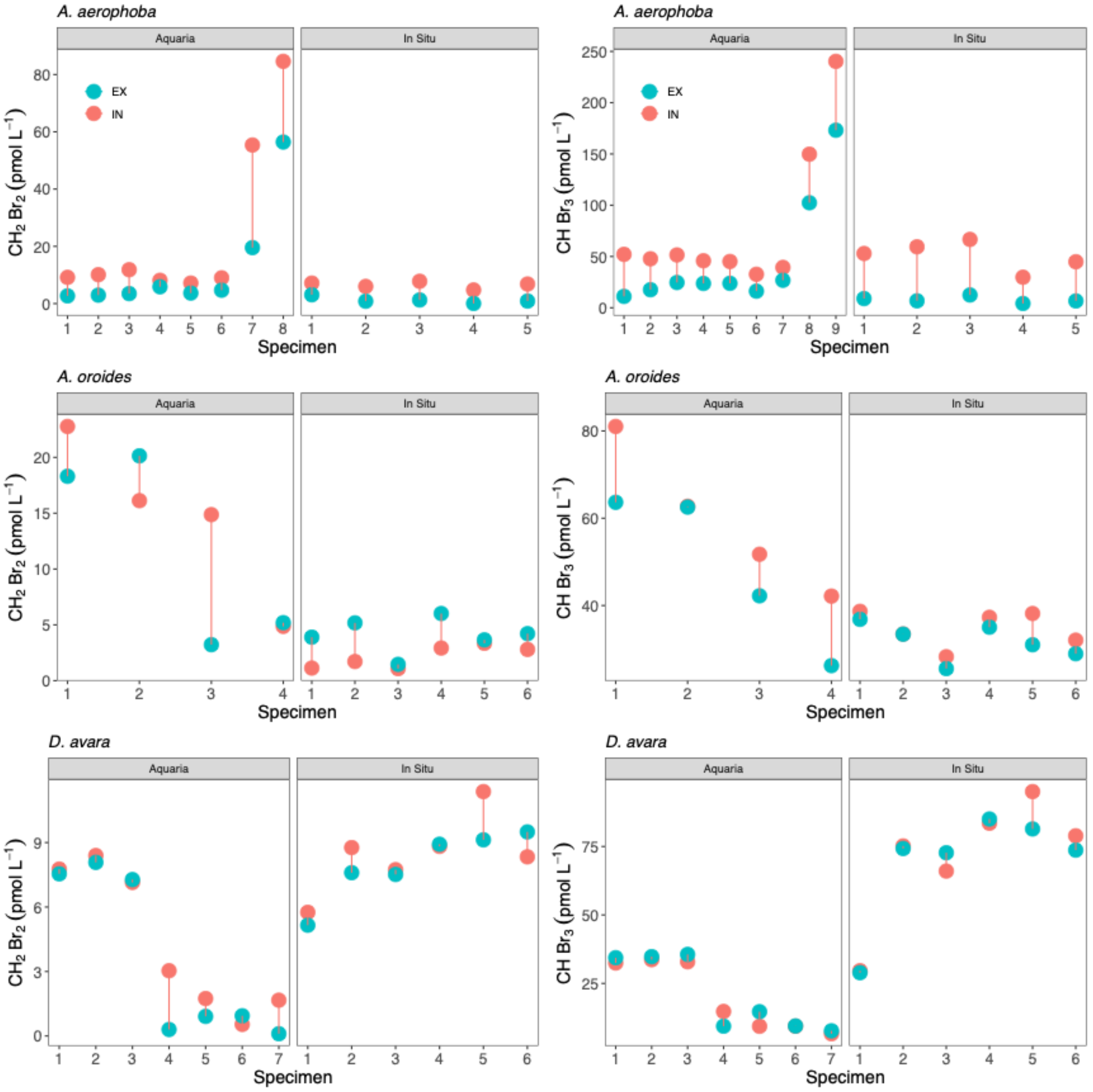

Figure S1: Detailed information about bromomethane concentrations in Exhaled (EX) and Inhaled (IN) waters in measurements conducted with the three sponges in aquaria and *in situ*.

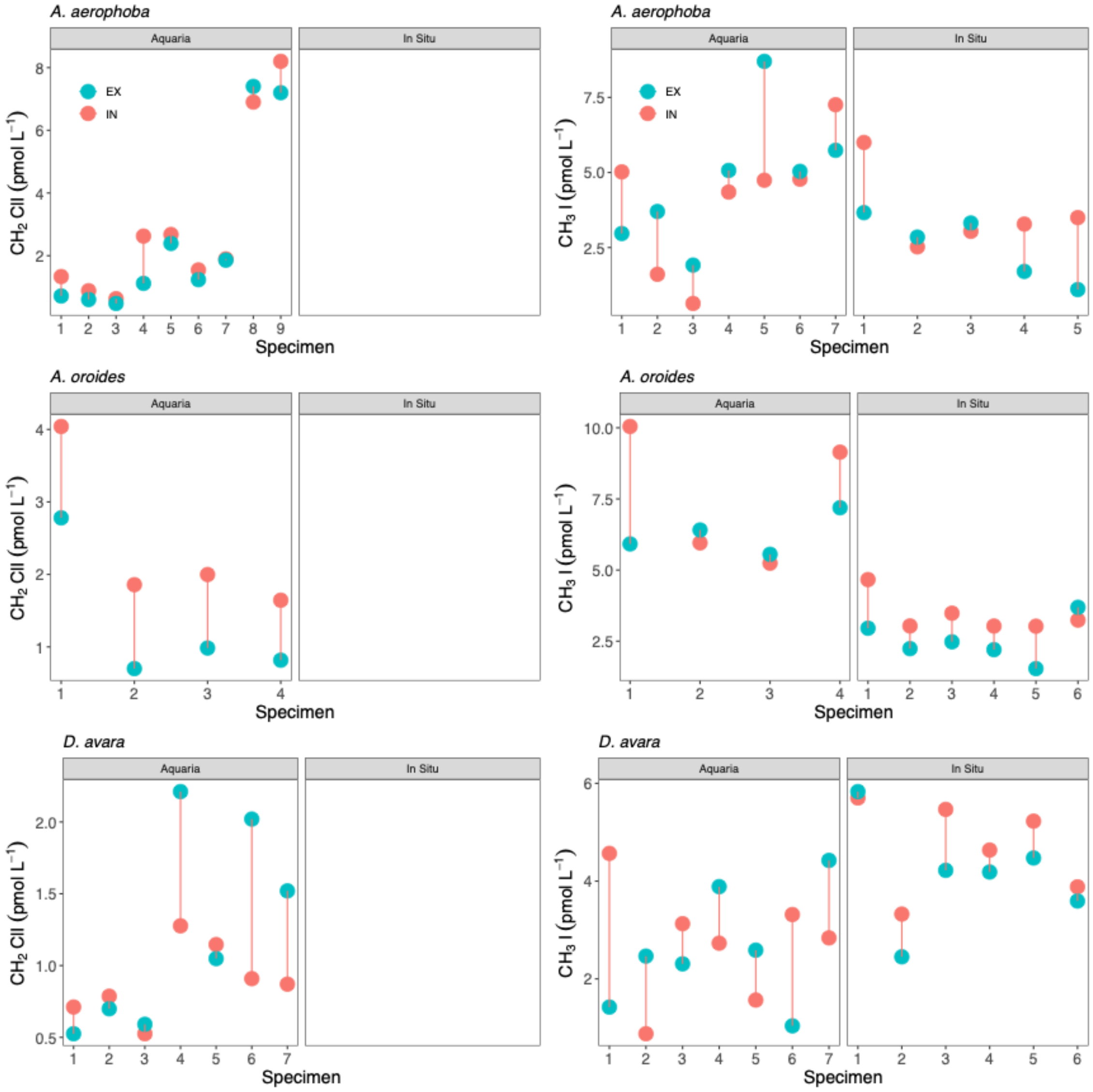

Figure S2: Detailed information about iodomethane concentrations in Exhaled (EX) and Inhaled (IN) waters in measurements conducted with the three sponges in aquaria and *in situ*.

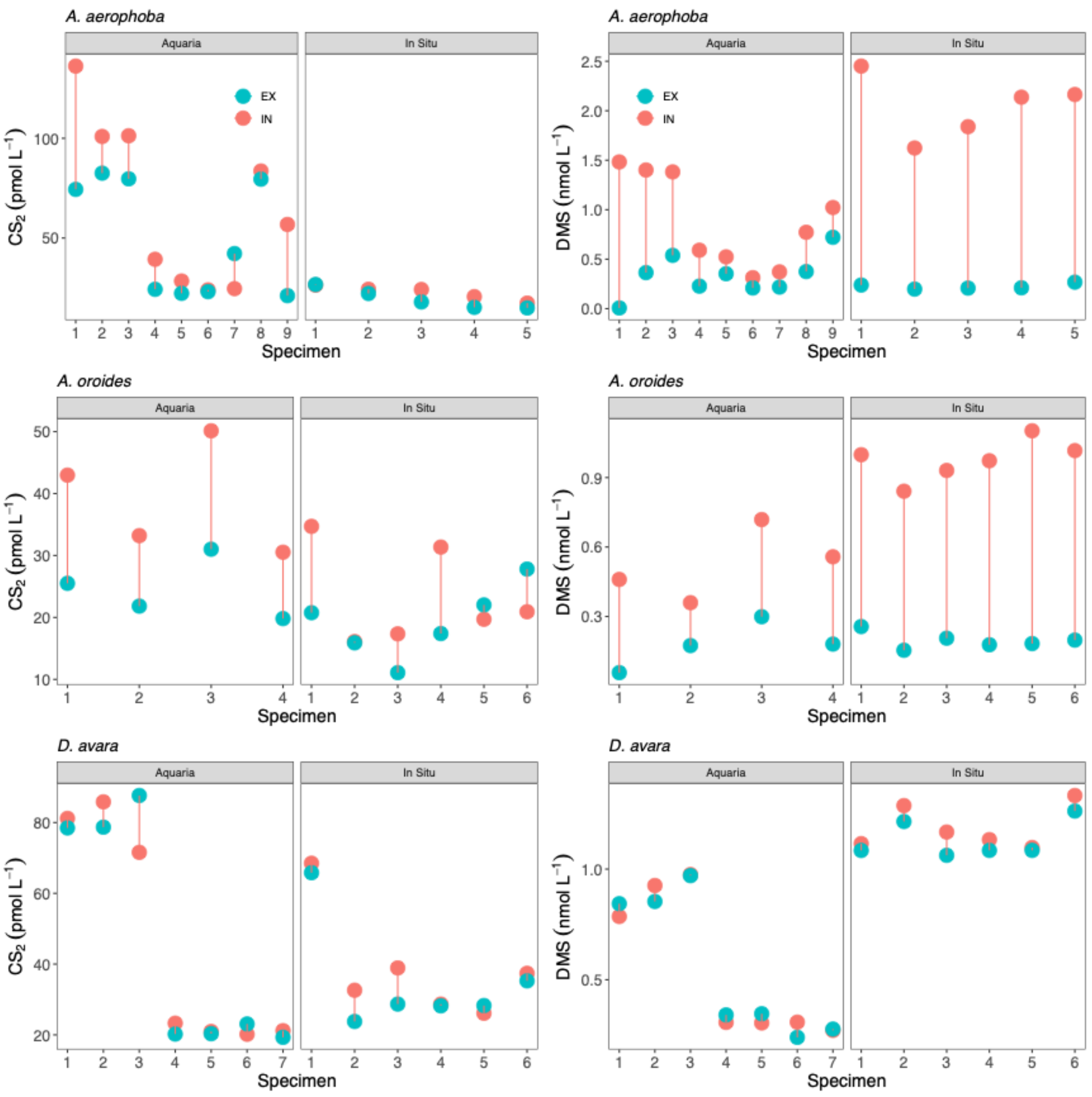

Figure S3: Detailed information about sulfur compound concentrations in Exhaled (EX) and Inhaled (IN) waters in measurements conducted with the three sponges in aquaria and *in situ*.

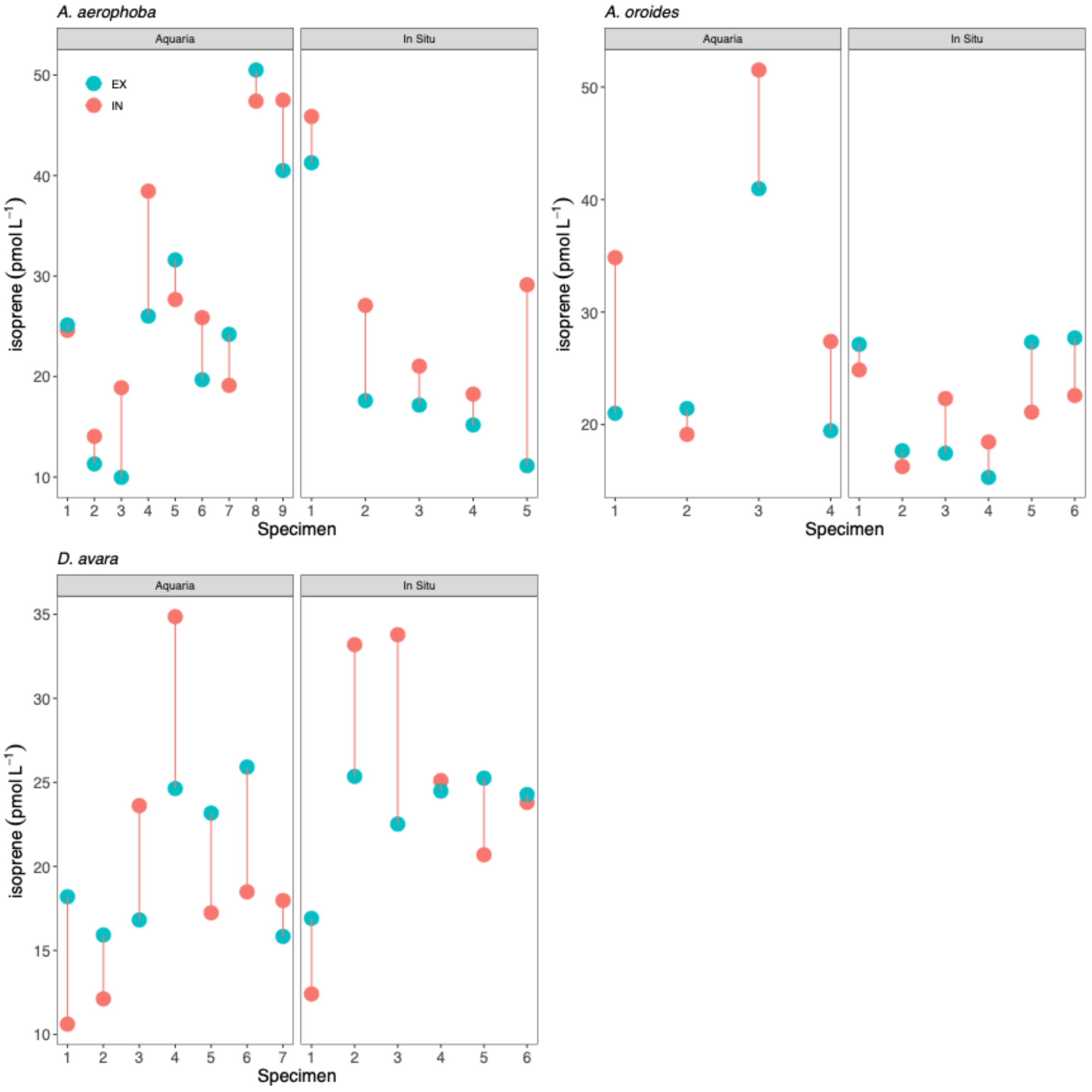

Figure S4: Detailed information about isoprene concentrations in Exhaled (EX) and Inhaled (IN) waters in measurements conducted with the three sponges in aquaria and *in situ*.
